# Bedarfe von Menschen mit schweren psychischen Erkrankungen

**DOI:** 10.1007/s00115-025-01835-5

**Published:** 2025-05-19

**Authors:** Johanna Breilmann, Andreas Allgöwer, Reinhold Kilian, Uta Gühne, Steffi G. Riedel-Heller, Alkomiet Hasan, Thomas Becker, Peter Falkai, Klemens Ajayi, Peter Brieger, Karel Frasch, Theresa Halms, Stephan Heres, Markus Jäger, Andreas Küthmann, Albert Putzhammer, Bertram Schneeweiß, Michael Schwarz, Markus Kösters

**Affiliations:** 1 Klinik für Psychiatrie und Psychotherapie II, Universität Ulm, BKH Günzburg, Lindenallee 2, 89312 Günzburg, Deutschland; 2 Institut für Epidemiologie und Med. Biometrie, Universität Ulm, Ulm, Deutschland; 3 Institut für Sozialmedizin, Arbeitsmedizin und Public Health, Universität Leipzig, Leipzig, Deutschland; 4 Klinik für Psychiatrie, Psychotherapie und Psychosomatik, Medizinische Fakultät, Universität Augsburg, BKH Augsburg, Augsburg, Deutschland; 5 Deutsches Zentrum für psychische Gesundheit (DZPG), Standort München/Augsburg, Augsburg, Deutschland; 6 Klinik und Poliklinik für Psychiatrie und Psychotherapie, Universitätsklinikum Leipzig, Leipzig, Deutschland; 7 Klinik für Psychiatrie und Psychotherapie, Universitätsklinikum München, München, Deutschland; 8 kbo-Isar-Amper Klinikum Region München, München, Haar, Taufkirchen, Deutschland; 9 Bezirkskrankenhaus Donauwörth, Donauwörth, Deutschland; 10 Bezirkskrankenhaus Kempten, Kempten, Deutschland; 11 Bezirkskrankenhaus Memmingen, Memmingen, Deutschland; 12 Bezirkskrankenhaus Kaufbeuren, Kaufbeuren, Deutschland; 13 Zentrum für Evidenzbasierte Gesundheitsversorgung, Universitätsklinikum Dresden und Medizinische Fakultät Carl Gustav Carus, Technische Universität Dresden, Dresden, Deutschland

**Keywords:** Funktionseinschränkungen, Psychiatrie, Bedarfsdeckung, Professionelle Unterstützung, Informelle Unterstützung, Functional limitations, Psychiatry, Health services needs, Health care providers, Informal caregivers

## Abstract

**Hintergrund:**

Aufgrund krankheitsbedingter Funktionseinschränkungen sind viele Menschen mit schweren psychischen Erkrankungen auf Unterstützung und Hilfe im Alltag angewiesen, um ein möglichst selbstständiges und gutes Leben führen zu können.

**Ziel der Arbeit:**

Die Studie untersuchte die Bedarfe, die Bedarfsdeckung sowie deren Einflussfaktoren bei Menschen mit schweren psychischen Erkrankungen in Deutschland.

**Methodik:**

Eingeschlossen wurden Menschen mit schweren psychischen Erkrankungen. Die Bedarfe und Bedarfsdeckung sowie die potenziellen Einflussfaktoren wurden mittels Camberwell Assessment of Need (CAN) und Client Sociodemographic and Service Receipt Inventory (CSSRI) erfasst und deskriptiv sowie explorativ analysiert.

**Ergebnisse:**

Im Mittel haben die Betroffenen 6,4 Bedarfe, von denen im Schnitt 40,6 % gedeckt sind. Fast alle Betroffenen (98 %) benötigen professionelle Unterstützung, die jedoch aus der Perspektive der Betroffenen unzureichend ist (je nach Themenbereich 9–86 %). Viele Betroffene erhalten zudem Unterstützung durch Angehörige (je nach Themenbereich 7–57 %). Die Faktoren Diagnose, Alter, Funktionalität, Haushaltseinkommen und Wohnsituation zeigen einen Einfluss auf die Bedarfszahl und -deckung.

**Diskussion:**

Den Ergebnissen lässt sich entnehmen, dass die Betroffenen vielfältige und komplexe Bedarfe haben, die jedoch unvollständig gedeckt sind. Insbesondere die Unterstützung durch professionelle Dienste wird als unzureichend wahrgenommen.

**Zusatzmaterial online:**

Zusätzliche Informationen sind in der Online-Version dieses Artikels (10.1007/s00115-025-01835-5) enthalten.

## Abstract (en)

**Background:**

Due to illness-related functional limitations, a significant proportion of individuals with severe mental illness are dependent on external assistance to navigate their daily lives and achieve an optimal level of independence and wellbeing.

**Objectives:**

The present study sought to investigate the needs, the coverage of needs and the influencing factors among people with severe mental illness in Germany.

**Methods:**

The study included patients diagnosed with severe mental illness. The met and unmet needs, as well as the potential influencing factors, were collected using Camberwell Assessment of Need (CAN) and Client Sociodemographic and Service Receipt Inventory (CSSRI) and subsequently analysed descriptively and exploratively.

**Results:**

The mean number of reported needs was 6.4, with an average of 40.6% of these needs being met. The vast majority of patients (98%) require professional support, which is, from their perspective, inadequate (9–86% depending on the area concerned). Additionally, many patients also receive support from relatives (7–57% depending on the area concerned). The diagnosis, age, functioning, household income, and housing situation have an influence on the number and coverage of needs.

**Conclusions:**

The findings indicate that patients have diverse and complex needs, which are not fully met. Notably, the support provided by professional services is perceived as inadequate.

Menschen mit schweren psychischen Erkrankungen haben oftmals vielfältige Bedarfe hinsichtlich der Bewältigung ihres Alltags und sind auf entsprechende Unterstützung angewiesen, um an der Gesellschaft teilhaben zu können. In diesem Beitrag wird die aktuelle Bedarfssituation von Menschen mit schweren psychischen Erkrankungen, die benötigte und erhaltene Unterstützung sowie Einflussfaktoren auf die Bedarfe dargestellt.

## Hintergrund

Viele Menschen mit schweren psychischen Erkrankungen^1^ sind aufgrund von Funktionseinschränkungen auf Unterstützung im Alltag angewiesen, um ein möglichst selbstständiges Leben führen zu können. Diese sogenannten „Bedarfe“ sind oft komplex und betreffen u. a. die Bewältigung des Alltags, soziale und wirtschaftliche Belange sowie Zugang zu Bildung, Beschäftigung und Wohnraum [1, 8, 10, 11]. Die Deckung der individuellen Bedarfe ist ein wichtiger Faktor für die Betroffenen, um sich befähigt zu fühlen, die Wahl und Kontrolle über ihr Leben zu haben und in die Gemeinschaft integriert zu sein [10]. Ungedeckte Bedarfe dagegen verringern die Chance auf Genesung und führen zu verminderter Lebensqualität [10].

Mithilfe von Fragebögen wie dem Camberwell Assessment of Need (CAN) [19] können in Studien die Bedarfe und Bedarfsdeckung von Patientengruppen erfasst und beschrieben werden. Insbesondere Anfang der 2000er-Jahre sind zahlreiche Publikationen zu den Bedarfen psychisch kranker Menschen in Europa (inkl. Deutschland), den USA und Australien entstanden. Da die Bedarfe und Bedarfsdeckung der Betroffenen vom jeweiligen Versorgungssystem der Länder abhängen, ist eine Übertragbarkeit von Ergebnissen zwischen den verschiedenen Ländern nur bedingt möglich. Die letzte Studie aus Deutschland, die die Bedarfe von Menschen mit schweren psychischen Erkrankungen mit dem CAN erfasste, stammt aus dem Jahr 2017 [17] und berichtet eine durchschnittliche Bedarfszahl von 4,7 Bedarfen. Das Ergebnis steht im Kontrast zu anderen vorherigen Studien aus Deutschland (z. B. [1]) und anderen europäischen Ländern (z. B. [16, 26]), in denen im Schnitt ca. 6 Bedarfe erfasst wurden.

Neben der Anzahl an Bedarfen erfasst der CAN auch, von wem und in welchem Ausmaß Betroffene Unterstützung erhalten. Hier zeigt sich ein Defizit der bisherigen Studien, v. a. aus Deutschland, in denen die Unterstützung bisher unzureichend analysiert wurde [1, 13, 17].

Ergänzend stellt die Identifikation von Einflussfaktoren auf die Bedarfe einen wichtigen Schritt zur Optimierung der Planung und Durchführung der Versorgung der Betroffenen dar [10]. Bisherige Analysen zu Einflussfaktoren auf die Anzahl an Bedarfen ergaben jedoch unterschiedliche Ergebnisse. So wurden Zusammenhänge zwischen der Anzahl an Bedarfen und dem Alter [4, 9, 29], der Diagnose [1, 27, 29], der Funktionalität [16, 23, 29] und dem Geschlecht [4, 23] beobachtet, allerdings oft mit entgegengesetzten Richtungen. So besteht z. B. Uneinigkeit darüber, ob Menschen mit Depression [1, 27] oder Menschen mit Schizophrenie [29] mehr Bedarfe haben. In anderen Studien konnten Zusammenhänge dieser Art nicht beobachtet werden [4, 28].

Aufgrund des Alters der vorherigen Studien und der bisher nur unvollständigen Auswertung des CAN soll in der vorliegenden Arbeit ein aktualisiertes und ausführliches Bild der derzeitigen Bedarfe und der Bedarfsdeckung von Menschen mit schweren psychischen Erkrankungen in Deutschland (Bayern) gezeichnet werden. Ziel dieser Studie ist daher, zum einen die Bedarfe, die Bedarfsdeckung und die benötigte und erhaltene Unterstützung bei Menschen mit schweren psychischen Erkrankungen zu analysieren, zum anderen sollen Faktoren, die mit einer höheren Anzahl an Bedarfen assoziiert sind, identifiziert werden.

## Methodik

Für die vorliegende Sekundäranalyse wurden Daten einer Querschnittsstudie (siehe Studienprotokoll Breilmann et al. [2]) verwendet. Die Methoden, Analysen und Ergebnisse sind gemäß STROBE [5] berichtet.

### Setting

Die Rekrutierung der Probanden und Probandinnen, das Screening und die Datenerhebung erfolgte von März bis September 2019 in 10 Kliniken, die die Pflichtversorgung für Menschen mit psychischen Erkrankungen in Oberbayern und Schwaben sicherstellen (Details siehe [2]).

### Stichprobe

Es wurden stationäre und tagesklinische Patienten und Patientinnen (alle Geschlechter, 18–65 Jahre alt, einwilligungsfähig) eingeschlossen, die eine schwere psychische Störung im Sinne der S3-Leitlinie „Psychosoziale Therapien bei schweren psychischen Störungen“ [7] haben (Krankheitsdauer ≥ 2 Jahre, erhebliche Einschränkungen der Aktivitäten des täglichen Lebens und des sozialen Funktionsniveaus; [7]). Abweichend von der S3-Leitlinie wurde die Erhebung auf Menschen mit Schizophrenie, schizotypen und wahnhaften Störungen (ICD-10 F2x) sowie mit affektiven Störungen (ICD-10 F3x) beschränkt, da diese die häufigsten Diagnosen innerhalb der schweren psychischen Erkrankungen darstellen. Als Kriterien für eine schwere psychische Erkrankung wurden als Schwellenwerte definiert: 1) ein „Global Assessment of Functioning“ (GAF) von ≤ 60 und 2) ein „Health of the Nation Outcome Scales“(HoNOS)-Wert von (a) ≥ 2 auf einem der Items der Subskala für symptomatische Probleme (Punkt 6, 7 und 8) und eine Punktzahl von ≥ 2 auf jedem der 4 Items der Subskala für soziale Probleme (Punkte 9, 10, 11 und 12), oder (b) eine Punktzahl von ≥ 3 bei mindestens einem dieser Items (9, 10, 11 oder 12). Darüber hinaus wurde die Erkrankungsdauer (> 2 Jahre) erfasst.

### Messungen

Geeignete Personen wurden vom Klinikpersonal zur Teilnahme an der Studie vorgeschlagen. Bei Zustimmung wurde ein Screening mit dem Global Assessment of Functioning (GAF) und dem Health of the Nation Outcome Scales (HoNOS) durch das Studienpersonal durchgeführt, um Personen mit schweren psychischen Erkrankungen zu identifizieren. Eingeschlossene Personen wurden vom Studienpersonal per Fragebogen befragt. Für die vorliegende Analyse wurden folgende Daten erhoben:

1. Die Bedarfe und die benötigte und erhaltene Unterstützung wurden aus Betroffenenperspektive anhand der deutschen Fassung des Camberwell Assessment of Need (CAN-EU; [13]) erfasst. Dieser bewertet 23 Themenbereiche hinsichtlich des Fehlens eines Bedarfs (0), des Vorhandenseins eines gedeckten Bedarfs (1) oder eines ungedeckten Bedarfs (2; siehe Supplement-Tab. A1). Für die Gesamtanzahl der Bedarfe wurden die Anzahl der Themenbereiche summiert, die einen Bedarf (ungedeckt/gedeckt) aufwiesen. Die benötigte und erhaltene Hilfe wurde auf einer Skala von 0 (= keine Hilfe) bis 3 (= viel Hilfe) erfasst und für die Analyse zu einer binären Variable mit 0 (= keine Hilfe) und 1 (Hilfe erhalten = Skalenwerte 1–3) zusammengefasst.
2. Die Charakteristika der Teilnehmenden (Geschlecht, Alter, etc.) wurden mit dem Client Sociodemographic and Service Receipt Inventory (CSSRI; [22]) erfasst. Die Diagnosen wurden den Krankenakten entnommen.

### Variablen

Folgende Faktoren wurden hinsichtlich des Einflusses auf die Bedarfe untersucht: Geschlecht (Mann vs. Frau), Alter, Diagnose (Schizophrenie vs. Depression vs. bipolare Störung), Wohnort (> 20.000 Einwohner vs. ≤ 20.000 Einwohner), Migrationshintergrund (ja vs. nein), Funktionsniveau (GAF), Erkrankungsdauer, Partnerbeziehung (in Partnerschaft vs. alleinstehend), Schulabschluss (kein/Förder‑/Hauptschulabschluss vs. Realschulabschluss vs. [Fach-]Abitur), Haushaltsnettoeinkommen/Monat (< 1000 € vs. 1000–2000 € vs. > 2000 €), Wohnsituation (selbstständig wohnend vs. betreut wohnend vs. wohnungslos).

### Stichprobengröße

Es wurde a priori eine Stichprobe von ca. 500 Teilnehmenden geplant. Da die Studie als deskriptive und explorative Studie geplant wurde, wurde keine Fallzahlkalkulation durchgeführt.

### Statistische Methoden

Die CAN-Auswertung folgte dem zugehörigen Manual [25]. Kategoriale Variablen wurden als absolute und relative Häufigkeiten, kontinuierliche Variablen als Mittelwert (M) und Standardabweichung (SD) dargestellt. Einflussfaktoren auf die Anzahl der Bedarfe wurden durch multiple lineare Regressionsanalyse (Einschlussmethode) untersucht. Für alle verwendeten Tests wurden die Modellannahmen überprüft und das statistischen Signifikanzlevel auf *p* < 0,05 festgelegt. Aufgrund des explorativen Charakters wurde keine α‑Adjustierung durchgeführt. Die statische Auswertung erfolgte mittels IBM SPSS Statistics für Windows, Version 28.0 (IBM Corp., Armonk, N.Y., USA).

## Ergebnisse

### Studienteilnehmer

**Abb. 1:**
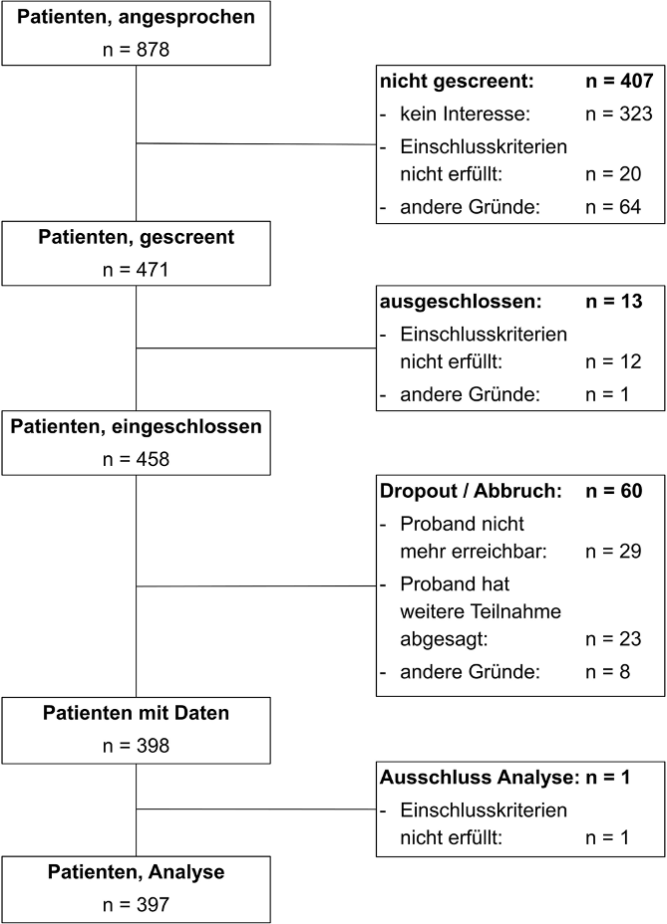
Flow-Chart Patientenrekrutierung

Für die Analysen konnten die Daten von 397 Personen verwendet werden. Details zum Patientenfluss können Abb. 1 entnommen werden. Die Charakteristika der Studienteilnehmer sind in Tab. 1 dargestellt.

**Tab. 1:** Charakteristika der Stichprobe (*n* = 397)

	*n* (%)/M (SD)
*Geschlecht, n (%)*	
Männer	171 (43,1)
Frauen	226 (56,9)
*Alter [Jahre], M (SD) (n* *=* *395)*	42,7 (13,2)
*Diagnose, n (%)*	
Depression (F32, F33)	232 (58,4)
Schizophrenie (F2x)	128 (32,2)
Bipolare Störung (F30, F31)	37 (9,3)
*Wohnort, n (%) (n* *=* *395)*	
≤ 20.000 Einwohner	162 (41,0)
> 20.000 Einwohner	233 (59,0)
*Migrationshintergrund, n (%) (n* *=* *393)*	
Nein	321 (81,7)
Ja	72 (18,3)
*GAF, M (SD) (n* *=* *390)*	42,3 (9,8)
*Erkrankungsdauer [Jahre], M (SD) (n* *=* *368)*	15,4 (10,9)
*Partnerbeziehung, n (%) (n* *=* *381)*	
In Partnerschaft	159 (41,7)
Alleinstehend	222 (58,3)
*Schulabschluss, n (%) (n* *=* *395)*	
Kein/Förder‑/Hauptschulabschluss	153 (38,7)
Realschulabschluss	114 (28,9)
(Fach‑)Abitur	128 (32,4)
*Haushaltsnettoeinkommen/Monat, n (%) (n* *=* *354)*	
< 1000 €	112 (31,6)
1000–2000 €	113 (31,9)
> 2000 €	129 (36,5)
*Wohnsituation, n (%) (n* *=* *395)*	
Selbstständig wohnend	341 (86,3)
Betreut wohnend	38 (9,6)
Wohnungslos	16 (4,1)

*M* Mittelwert, *SD* Standardabweichung

### Gesamtbedarf und Bedarfsdeckung

Insgesamt 97 % (385/397) der Befragten gaben an, Bedarfe zu haben. Im Mittel wurden 6,4 Bedarfe (SD 2,9) genannt, von denen 40,6 % (M 2,6; SD 2,0) gedeckt und 59,4 % (M 3,8; SD 2,6) ungedeckt waren.

Mit zunehmendem Alter berichteten die Betroffenen von weniger Bedarfen: je 20 Jahre ca. 1 Bedarf (*p* = 0,001) und ca. 0,5 ungedeckte Bedarfe weniger (*p* = 0,046; Tab. 2). Personen mit Depression gaben im Schnitt ca. 1,5 Bedarfe (*p* < 0,001 bzw. *p* = 0,003) und ca. 1 ungedeckten Bedarf (*p* < 0,001 bzw. *p* = 0,019) mehr an als Personen mit anderen Diagnosen. Des Weiteren gaben Personen mit niedriger psychosozialer Funktionalität je Abnahme des GAF-Werts um 15 Einheiten ca. 1 Bedarf und 1 ungedeckten Bedarf (je *p* < 0,001) mehr an. Betroffene, die im betreuten Wohnen leben, gaben 1 Bedarf (*p* = 0,049) und 1,5 gedeckte Bedarfe (*p* < 0,001) mehr an als selbstständig wohnende Betroffene. Betroffene mit niedrigem monatlichem Haushaltsnettoeinkommen (< 1000 €) gaben rund 1 ungedeckten Bedarf mehr (*p* = 0,025) an als Personen mit hohem Einkommen (> 2000 €).

**Tab. 2:** Einflussfaktoren auf die Anzahl an Bedarfen^a^ (*n* = 302)

	Anzahl Bedarfe			Anzahl gedeckter Bedarfe			Anzahl ungedeckter Bedarfe		
	B	SE	*P*	B	SE	*P*	B	SE	*P*
Mann^b^	−0,44	0,32	0,171	−0,36	0,23	0,124	−0,08	0,31	0,789
Alter [Jahre]	−0,04	0,01	*0,001*	−0,02	0,01	0,067	−0,03	0,01	0,046
Schizophrenie^b^	−1,34	0,35	*<* *0,001*	−0,17	0,26	0,510	−1,18	0,34	*<* *0,001*
Bipolare Störung^b^	−1,51	0,51	*0,003*	−0,34	0,37	0,361	−1,17	0,50	*0,019*
> 20.000 Einwohner^b^	−0,21	0,34	0,521	−0,10	0,24	0,676	−0,12	0,33	0,726
Migrationshintergrund^b^	−0,09	0,42	0,825	−0,14	0,30	0,646	0,05	0,41	0,909
GAF	−0,06	0,02	*<* *0,001*	−0,01	0,01	0,346	−0,05	0,02	*<* *0,001*
Erkrankungsdauer [Jahre]	0,02	0,02	0,347	−0,01	0,01	0,660	0,02	0,02	0,196
In Partnerschaft^b^	−0,14	0,35	0,695	−0,05	0,25	0,858	−0,09	0,34	0,787
Realschulabschluss^b^	−0,38	0,37	0,303	−0,06	0,27	0,837	−0,33	0,36	0,364
(Fach‑)Abitur^2^	−0,29	0,39	0,452	0,25	0,28	0,373	−0,55	0,38	0,152
1000–2000 €/Monat^2^	−0,56	0,42	0,182	−0,06	0,30	0,835	−0,50	0,41	0,222
> 2000 €/Monat^b^	−0,77	0,46	0,095	0,24	0,33	0,476	−1,01	0,45	*0,025*
Betreutes Wohnen^b^	1,07	0,54	*0,049*	1,55	0,39	*<* *0,001*	−0,48	0,53	0,365
Wohnungslos^b^	1,15	0,82	0,162	0,75	0,59	0,209	0,40	0,80	0,613
Gesamtmodell	F (15, 286) = 5,308; *p* *<* *0,001*; korr. R^2^ = 0,177			F (15, 286) = 2,273; *p* *=* *0,005*; korr. R^2^ = 0,060			F (15, 286) = 3,224; *p* *<* *0,001*; korr. R^2^ = 0,100		

*B* Regressionskoeffizient, *SE* Standardfehler

^a^Ergebnisse der multiplen linearen Regressionsanalyse

^b^Dummy-Variable: ja = 1; nein (Frau; Depression; Wohnort ≤ 20.000 Einwohner; kein Migrationshintergrund; alleinstehend; kein/Förder‑/Hauptschulabschluss; Haushaltsnettoeinkommen < 1000 €/Monat; selbstständiges Wohnen) = 0

### Einzelne Bedarfe und Unterstützung

**Abb. 2:**
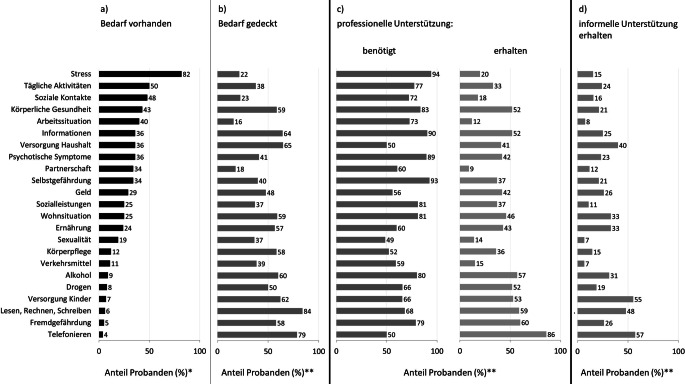
Relativer Anteil (%) an Menschen mit schweren psychischen Erkrankungen mit (nach eigener Auskunft): *a* Bedarf im jeweiligen Themenbereich; *b* gedecktem Bedarf; *c* benötigter/erhaltener professioneller Unterstützung; *d* erhaltener informeller Unterstützung. *Asterisk* *N* = 397; *Doppelasterisk* Betroffene, die im jeweiligen Themenbereich einen Bedarf angaben. (Erläuterungen zu den jeweiligen Themenbereichen befinden sich in Supplement-Tab. A1)

Betroffene gaben am häufigsten Bedarfe in den Bereichen Stress, tägliche Aktivitäten, soziale Kontakte, körperliche Gesundheit und Arbeitssituation an (Abb. 2a). Am seltensten wurden Bedarfe in den Bereichen Telefonieren, Fremdgefährdung, Lesen, Rechnen, Schreiben, Versorgung der Kinder und Drogen genannt. Je nach Themenbereich gaben 16–84 % der Betroffenen eine Bedarfsdeckung, v. a. in den Bereichen Lesen, Rechnen, Schreiben und Telefonieren, an. In den Bereichen Arbeitssituation und Partnerschaft sind die Bedarfe am wenigsten gedeckt (Abb. 2b).

Insgesamt 98 % (376/385) der Betroffenen benötigen bei mindestens einem ihrer Bedarfe professionelle Unterstützung (je nach Themenbereich 49–94 %; Abb. 2c). Diese Hilfe erhielten jedoch nach eigenen Angaben nur 9–86 % der Betroffenen (Abb. 2c), wobei in allen Bereichen (außer Telefonieren), deutlich weniger professionelle Unterstützung geleistet wurde als benötigt. Die größten Differenzen bestehen in den Bereichen, in denen viele Betroffene einen Bedarf äußerten (Stress, Arbeitssituation, Selbstgefährdung, soziale Kontakte), was sich in den niedrigen Zahlen der Bedarfsdeckung widerspiegelt (16–40 %; Abb. 2b). In Bereichen mit nur wenigen Bedarfen (Telefonieren, Fremdgefährdung, etc.) ist die Bedarfsdeckung dagegen höher (58–84 %; Abb. 2b).

Unterstützung durch Familie und Bekannte (informelle Hilfe) berichteten je nach Themenbereich 7–57 % der Betroffenen, wobei am häufigsten in den Bereichen Telefonieren, Versorgung der Kinder und Lesen, Rechnen, Schreiben Hilfe geleistet wurde (Abb. 2d).

Erläuterungen zu den jeweiligen Themenbereichen befinden sich in Supplement-Tab. A1.

## Diskussion

Nahezu alle Probanden und Probandinnen dieser Studie (97 %) berichteten Unterstützungsbedarfe, insbesondere in den Bereichen Stress, tägliche Aktivitäten, soziale Kontakte, körperliche Gesundheit und Arbeitssituation, ähnlich wie in einer anderen deutschen Studie [1]. Im Schnitt wurden 6,4 Bedarfe angegeben, von denen jedoch im Mittel lediglich 41 % gedeckt waren. Dies steht im Gegensatz zu früheren Studien, die im Zeitraum von 2001 bis 2017 einen von den Betroffenen berichteten Bedarfsdeckungsgrad von 51–67 % ausweisen [1, 13, 17]. Dies deutet auf eine heute als geringer wahrgenommene Bedarfsdeckung hin, die auch mit einer von den Betroffenen als unzureichend bewerteten professionellen Unterstützung einhergeht. Fast alle Betroffenen dieser Studie (98 %) benötigen nach eigenen Angaben bei mindestens einem Bedarf professionelle Unterstützung. Diese Unterstützung erhalten jedoch je nach Themenbereich nur 9–86 % der Betroffenen, während im Jahr 2000 diese noch mit 29–100 % berichtet wurde [11].

Die unterschiedlichen Ergebnisse sind möglicherweise auf einen höheren Anteil an Personen mit Schizophrenie in drei Studien (55–100 % vs. 32 %; [1, 11, 13]) im Vergleich zur vorliegenden Studie zurückzuführen. Auch war der GAF-Wert in einer Studie [13] höher als in dieser Studie (54,6 vs. 42,3). Möglicherweise sind die subjektiv wahrgenommene niedrige Bedarfsdeckung und Unterstützung jedoch auch Ausdruck einer gestiegenen Komplexität bzw. höheren Anforderung an eine moderne Versorgung psychisch erkrankter Menschen. Die S3-Leitlinie „Psychosoziale Therapien bei schweren psychischen Störungen“ [7] legt seit dem Jahr 2013 Empfehlungen für die Bereiche Arbeit, Training von Alltags- und sozialen Fertigkeiten und Ansätze zum Selbstmanagement im Umgang mit Stress vor. Eine Erhebung der regionalen Versorgungsstrukturen aus dem Jahr 2019 zeigte jedoch, dass insbesondere in den Bereichen Home Treatment/Stäb, psychiatrische häusliche Krankenpflege, ambulante Ergo‑, Kreativ- und Soziotherapie und Supported Employment nur wenige Angebote existieren [3], wodurch die Umsetzung der Empfehlungen erschwert bleibt.

Neben der professionellen Unterstützung ist die Hilfe durch Angehörige und Freunde die zweite große Säule der Hilfeleistung. Je nach Themenbereich erhalten allerdings nur 7–57 % der Betroffenen diese Unterstützung und dies am ehesten in alltäglichen Bereichen wie Telefonieren, Versorgung von Kindern und Lesen, Rechnen, Schreiben. Im Jahr 2000 lag die Unterstützung noch bei 46–83 % [11]. Diese Entwicklung spiegelt wahrscheinlich einen gesellschaftlichen Trend wider, wonach die Unterstützung innerhalb von Familiennetzwerken und Bekanntenkreisen abnimmt [15]. Zu dem liegt der Fokus der Hilfe zunehmend auf weniger aufwendigen Tätigkeiten (z. B. Hilfe im Haushalt und Begleitung) [15].

Jüngere Betroffene und Personen mit Depression, mit niedrigerer psychosozialer Funktionalität und mit niedrigerem monatlichem Haushaltsnettoeinkommen gaben in der Befragung höhere Bedarfe, und damit einhergehend höhere ungedeckte Bedarfe, an. Diese Patientengruppen scheinen daher bei der Unterstützung besonderer Beachtung zu bedürfen. Betroffene, die im betreuten Wohnen leben, gaben ebenfalls mehr Bedarfe als selbstständig wohnende Personen an, nannten aber gleichzeitig auch mehr gedeckte Bedarfe. Zunächst kontraintuitiv ist, dass Personen mit Schizophrenie weniger Bedarfe berichteten als Personen mit Depression, was auch in anderen Studien beobachtet wurde [1, 13, 27]. Als Ursachen hierfür werden eine geringere Krankheitseinsicht [12], eine höhere selbst eingeschätzte Lebensqualität [21] und eine höhere Resilienz [18] von Betroffenen mit Schizophrenie vermutet. Eine ergänzende Einschätzung der Bedarfssituation durch die Behandelnden kann weitere Erkenntnisse ergeben, wobei sich jedoch gezeigt hat, dass nur die Deckung der von den Betroffenen selbst angegebenen Bedarfe ihre Lebensqualität verbessert [14].

Bei Bemühungen um eine Verbesserung der Versorgungsrealität können (a) der Integrationsgrad von Versorgungsangeboten, (b) die Integration von Qualifikationen bzw. Berufsgruppen und (c) die Integration von Versorgungssystem und zivilgesellschaftlichen Initiativen erwogen werden. Lokale und regionale Versorgungssysteme mit flexiblen Übergängen zwischen verschiedenen Leistungen des SGB V (z. B. stationäre/teilstationäre Behandlung, StäB/Home Treatment, psychiatrische Institutsambulanz, fachärztliche und psychologisch-psychotherapeutische Praxis sowie ambulante psychiatrische Pflege) können eine bessere Annäherung an die Lebenssituation erkrankter Menschen leisten (sog. Regionalbudgets, Vereinbarungen mit Leistungserbringern nach § 64 SGB V; [24]). Eine gemeinsame konzeptuelle und fachpolitische Betonung lebenswelt- und bedarfsorientierter fachlicher Angebote zwischen den im Hilfesystem aktiven Berufsgruppen und ihren Fachverbänden und -gesellschaften könnte förderlich sein (vernetzte Teams, Suche nach neuen Vergütungsformen). Schließlich könnte die Nutzung von Kooperationsmöglichkeiten zwischen Anbietern von Gesundheitsleistungen (SGB V) und Hilfsangeboten komplementärer Träger, von Vereinen, Selbsthilfefirmen und psychosozialen Hilfsgemeinschaften (andere, diverse Kostenträgerschaften und Finanzierungsformen) hilfreich sein.

### Limitationen

Die Studie birgt ein Risiko für einen Selektionsbias hinsichtlich der Patientengruppe, da möglicherweise nur Personen teilgenommen haben, die sich gesund genug für die Teilnahme fühlten. Darüber hinaus wurden nur (teil-)stationäre Patienten und Patientinnen bayerischer Kliniken eingeschlossen, sodass die Ergebnisse ggf. nicht generalisierbar sind. Die Einschlusskriterien für die Probandengruppe orientierten sich in dieser Studie an der Definition für „schwere psychische Erkrankungen“ der S3-Leitlinie „Psychosoziale Therapien bei schweren psychischen Störungen“ [7], mit einer Beschränkung auf die Diagnosen ICD-10 F2x und ICD-10 F3x. Die Untersuchung ist somit nicht repräsentativ für Probandengruppen, bei der sich die Definition einer schweren psychischen Erkrankung an anderen Kriterien (vgl. [6]) orientiert. Die Befragung gibt des Weiteren ausschließlich die subjektive Perspektive der Betroffenen wieder und stellt keine Messung der real erhaltenen Unterstützung dar. Des Weiteren sollte beachtet werden, dass die simplifizierte Erfassung von Bedarfen keine Informationen darüber liefert, welche Art und Menge an Unterstützung tatsächlich erforderlich ist [20].

Die Erfassung der Bedarfsdeckung erfolgte in der vorliegenden Studie ausschließlich über Selbstberichte der Patienten und Patientinnen. Für zukünftigen Studien sollte geprüft werden, inwieweit die Erfassung der Bedarfsdeckung zudem mit Routinedaten ergänzt werden kann.

## Fazit für die Praxis

- Fast alle Betroffenen mit schweren psychischen Erkrankungen haben diverse Bedarfe, die derzeit nur unzureichend gedeckt sind. Frühere Studien zeigen eine höhere Bedarfsdeckung.
- Die Leistungen der professionellen Dienste werden als unzureichend wahrgenommen, was darauf hindeuten könnte, dass die Versorgung der Betroffenen nicht bedarfsgerecht erfolgt.
- Betroffene mit Depression, jüngeren Alters, mit niedriger psychosozialer Funktionalität und mit niedrigem monatlichem Haushaltsnettoeinkommen gaben mehr Bedarfe und mehr ungedeckte Bedarfe an. Auf diese Patientengruppen sollte daher bei der Unterstützung besonders geachtet werden.

## Supplementary Information

Supplement-Tab. A1.

## Data Availability

Aus Datenschutzgründen sind die im Rahmen der vorliegenden Studie generierten und analysierten Datensätze nicht öffentlich zugänglich, können aber bei angemessener Anfrage beim entsprechenden Autor angefordert werden.

## Footnotes

1 Im Sinne der Definition von „schweren psychischen Erkrankungen“ der S3-Leitlinie „Psychosoziale Therapien bei schweren psychischen Störungen“ [7]: „Menschen mit jeder psychiatrischen Diagnose, welche über längere Zeit, d. h. über mindestens zwei Jahre, Krankheitssymptome aufweisen bzw. in Behandlung sind, die mit erheblichen Auswirkungen auf die Aktivitäten des täglichen Lebens und das soziale Funktionsniveau einhergehen sowie häufig mit einer intensiven Inanspruchnahme des Behandlungs- und psychosozialen Hilfesystems verbunden sind.“ (Gühne et al. [7, S. 7f.]).
