# Supplementary material for: Needs of people with severe mental illness
Source: Nervenarzt. 2025 May 19;97(1):74–81. [Article in German] doi: 10.1007/s00115-025-01835-5 (PMC12808281; doi:10.1007/s00115-025-01835-5)
Supplement: Supplementary file 1 — Supplement-Tab. A1. [file 115_2025_1835_MOESM1_ESM.pdf]

Supplement zu Breilmann et al.: Bedarfe von Menschen mit schweren psychischen Erkrankungen

Supplement-eTab. 1: Themenbereiche und dazugehörige Fragestellung des Camberwell Assessment of Need-European Version (CAN-EU)[1]

Themenbereiche des CAN	Erläuterung (Fragestellung aus dem CAN)
**Wohnsituation**	HAT DIE PERSON GEGENWÄRTIG KEINE WOHNUNG / UNTERKUNFT?<br>*Wo leben Sie im Moment?*<br>*Was für eine Art Unterkunft ist das?*
**Ernährung**	HAT DIE PERSON PROBLEME, SICH AUSREICHEND ZU ERNÄHREN?<br>*Welche Art von Nahrung nehmen Sie zu sich?*<br>*Sind Sie in der Lage, Ihre Mahlzeiten selbst zuzubereiten und selbst einkaufen zu gehen?*
**Versorgung des Haushaltes**	HAT DIE PERSON PROBLEME, SICH UM DEN HAUSHALT ZU KÜMMERN?<br>*Sind Sie in der Lage, sich um Ihren Haushalt zu kümmern?*<br>*Hilft Ihnen jemand dabei?*
**Persönliche Körperpflege**	HAT DIE PERSON PROBLEME MIT DER KÖRPERPFLEGE?<br>*Haben Sie Probleme, sich sauber und ordentlich zu halten?*<br>*Müssen Sie an die Körperpflege erinnert werden? Durch wen?*
**Tägliche Aktivitäten**	HAT DIE PERSON PROBLEME BEI DER GESTALTUNG REGELMÄßIGER, ANGEMESSENER TAGESAKTIVITÄTEN?<br>*Wie verbringen Sie Ihren Tag?*<br>*Haben Sie genug zu tun?*
**Körperliche Gesundheit**	HAT DIE PERSON IRGENDEINE KÖRPERLICHE BEHINDERUNG ODER KÖRPERLICHE ERKRANKUNG?<br>*Wie fühlen Sie sich körperlich?*<br>*Werden Sie wegen irgendwelchen körperlichen Problemen von einem Arzt behandelt?*
**Psychotische Symptome**	HAT DIE PERSON IRGENDWELCHE PSYCHOTISCHE SYMPTOME (Z.B. WAHNVORSTELLUNGEN, HALLUZINA-TIONEN ODER FORMALE DENKSTÖRUNGEN)?<br>*Hören Sie manchmal Stimmen oder haben Sie Probleme mit Ihren Gedanken?*<br>*Bekommen Sie Medikamente oder Spritzen? Wofür sind diese?*
**Informationen (ü. Krankheit und Behandlung)**	HAT DIE PERSON KLARE MÜNDLICHE ODER SCHRIFTLICHE INFORMATIONEN ÜBER IHRE KRANKHEIT UND DEREN BEHANDLUNG ERHALTEN?<br>*Haben Sie klare Informationen über Ihre Medikamente oder andere Behandlungsformen erhalten?*<br>*Wie hilfreich waren diese Informationen für Sie?*
**Stress (seelischer Druck)**	LEIDET DIE PERSON GEGENWÄRTIG UNTER SEELISCHEM DRUCK?<br>*Haben Sie sich in letzter Zeit sehr traurig oder niedergeschlagen gefühlt?*<br>*Fühlten Sie sich ungewöhnlich ängstlich, oder fürchteten Sie sich vor etwas?*

**Selbstgefährdung**	IST DIE PERSON EINE GEFAHR FÜR SICH SELBST?<br>*Denken Sie manchmal daran, sich selbst etwas anzutun oder haben Sie sich tatsächlich selbst etwas angetan?*<br>*Bringen Sie sich selbst auf andere Weise in Gefahr?*
**Fremdgefährdung**	IST DIE PERSON TATSÄCHLICH ODER POTENTIELL EINE GEFAHR FÜR ANDERE MENSCHEN?<br>*Glauben Sie, dass Sie eine Gefahr für die Sicherheit anderer Personen sein könnten? Verlieren Sie gelegentlich Ihre Beherrschung und schlagen Sie dann jemanden?*
**Alkohol**	TRINKT DIE PERSON ÜBERMÄßIG ODER HAT SIE PROBLEME, IHREN ALKOHOLKONSUM ZU KONTROLLIEREN?<br>*Haben Sie Probleme mit Ihrem Alkoholkonsum?*<br>*Wünschten Sie sich, weniger Alkohol zu trinken?*
**Drogen**	LIEGT BEI DER PERSON EIN DROGENMISSBRAUCH VOR?<br>*Nehmen Sie irgendwelche Drogen oder Medikamente, die ihnen nicht verschrieben wurden?*<br>*Gibt es irgendwelche Drogen oder Medikamente, bei denen es Ihnen schwerfällt, mit der Einnahme aufzuhören?*
**soziale Kontakte**	BRAUCHT DIE PERSON UNTERSTÜTZUNG BEI SOZIALEN KONTAKTEN?<br>*Sind Sie zufrieden mit Ihren sozialen Aktivitäten?*<br>*Wünschen Sie sich, mehr Kontakt zu anderen Menschen zu haben?*
**Partnerschaft**	HAT DIE PERSON PROBLEME EINEN PARTNER ZU FINDEN ODER EINE ENGE BEZIEHUNG AUFRECHT ZU ERHALTEN?<br>*Haben Sie einen Partner?*<br>*Haben Sie Probleme in Ihrer Partnerschaft/Ehe?*
**Sexualität**	HAT DIE PERSON PROBLEME MIT IHREM SEXUALLEBEN?<br>*Wie ist Ihr Sexualleben?*
**Versorgung / Betreuung Kinder**	HAT DIE PERSON PROBLEME, SICH UM IHRE KINDER ZU KÜMMERN?<br>*Haben Sie Kinder, die jünger als 18 sind?*<br>*Haben Sie Probleme, sich um sie zu kümmern?*
**Grundkenntnisse Lesen, Rechnen, Schreiben**	MANGELT ES DER PERSON AN GRUNDKENNTNISSEN IM RECHNEN, LESEN UND SCHREIBEN?<br>*Haben Sie Probleme beim Lesen, Schreiben oder Verständnis der deutschen Sprache?*<br>*Können Sie Ihr Wechselgeld in einem Geschäft nachzählen?*
**Telefonieren**	HAT DIE PERSON PROBLEME, ZUGANG ZU EINEM TELEFON ZU BEKOMMEN ODER EIN TELEFON ZU BENUTZEN?<br>*Wissen Sie, wie man ein Telefon benutzt?*<br>*Ist es leicht für Sie ein Telefon zu finden, das Sie benutzen können?*
**Verkehrsmittel**	HAT DIE PERSON PROBLEME, ÖFFENTLICHE VERKEHRSMITTEL ZU BENUTZEN?<br>*Wie kommen Sie mit der Benutzung von Bus, Straßenbahn oder Zug zurecht? Haben Sie einen Freifahrtausweis?*
**Geld**	HAT DIE PERSON PROBLEME, IHR GELD EINZUTEILEN?<br>*Wie kommen Sie mit der Einteilung Ihres Geldes zurecht?*<br>*Sind Sie in der Lage, Ihre Rechnungen zu bezahlen?*

**Sozialleistungen**	ERHÄLT DIE PERSON WIRKLICH ALLE IHR ZUSTEHENDEN SOZIALLEISTUNGEN?<br>*Sind Sie sicher, dass Sie alle Ihnen zustehenden Geldbeträge erhalten?*
**Arbeitssituation**	HAT DIE PERSON GEGENWÄRTIG EINEN ANGEMESSENEN ARBEITSPLATZ?<br>*Wo arbeiten Sie zurzeit?*<br>*Entspricht dieser Arbeitsplatz Ihren gegenwärtigen Fähigkeiten?*

[1] Antwortmöglichkeiten: 0 = kein Problem; 1 = kein/geringes Problem, weil Hilfe geleistet wird; 2 = ernstes Problem; 9 = unbekannt.
